# Social approach and social vigilance are differentially regulated by oxytocin receptors in the nucleus accumbens

**DOI:** 10.1038/s41386-020-0657-4

**Published:** 2020-03-20

**Authors:** Alexia V. Williams, Natalia Duque-Wilckens, Stephanie Ramos-Maciel, Katharine L. Campi, Shanu K. Bhela, Christine K. Xu, Kenneth Jackson, Bice Chini, Patricia A. Pesavento, Brian C. Trainor

**Affiliations:** 1 Department of Psychology, University of California, Davis, CA USA; 2 Department of Pathology, Microbiology and Immunology, School of Veterinary Medicine, University of California, Davis, CA USA; 3 Isnstitute of Neuroscience of National Research Council and NEUROMI Milan Center for Neuroscience, University of Milano-Bicocca, Milan, Italy

**Keywords:** Stress and resilience, Neuroscience

## Abstract

Oxytocin is currently being considered as a novel therapeutic for anxiety disorders due to its ability to promote affiliative behaviors. In the nucleus accumbens (NAc) activation of oxytocin receptors (OTR) promotes social approach (time spent near an unfamiliar individual). Here, we show that stressful social experiences reduce the expression of NAc OTR mRNA, coinciding with decreases in social approach. Social stressors also increase social vigilance, characterized as orienting to an unfamiliar individual without approaching. Vigilance is a key component of behavioral inhibition, a personality trait that is a risk factor for anxiety disorders. To understand whether NAc OTR can modulate both social approach and vigilance, we use pharmacological approaches to assess the impact of activation or inhibition of NAc OTR downstream pathways on these behaviors. First, we show that in unstressed male and female California mice, inhibition of OTR by an unbiased antagonist (L-368,899) reduces social approach but does not induce social vigilance. Next, we show that infusion of Atosiban, an OTR-Gq antagonist/OTR-Gi agonist, has the same effect in unstressed females. Finally, we show that Carbetocin, a biased OTR-Gq agonist, increases social approach in stressed females while simultaneously inhibiting social vigilance. Taken together these data suggest that OTR in the NAc differentially modulate social approach and social vigilance, primarily through an OTR-Gq mechanism. Importantly, pharmacological inhibition of OTR alone is insufficient to induce vigilance in unstressed mice, suggesting that mechanisms modulating social approach may be distinct from mechanisms modulating social vigilance.

## Introduction

Avoidance of social situations is a hallmark symptom of a variety of psychiatric illnesses, including mood disorders, anxiety disorders [1], and several neurodevelopmental disorders such as autism [2, 3]. Although existing pharmacotherapeutics can help mitigate social avoidance in some individuals, for many these treatments prove insufficient. The neuropeptide oxytocin is being considered as a novel therapeutic target due to its ability to promote affiliative behaviors. When administered intranasally, oxytocin often increases various aspects of social behaviors [4–8]. However, other reports find that intranasal oxytocin treatment can have very different effects, especially in situations where an undercurrent of stress or threat exists [9, 10]. For example, in some cases, intranasal oxytocin has been shown to increase antagonistic social interactions in both humans [11, 12] and female Wistar rats [13–15]. Taken together this supports the idea that the impacts of oxytocin on social behavior are complex and differ based on context [16, 17]. Actions of oxytocin appear to be circuit-specific [17], which may be one possible mechanism for its context-dependent actions.

When acting within the mesolimbic dopamine system, oxytocin can promote several forms of social behavior [18–22]. Within the nucleus accumbens core (NAc), oxytocin promotes the formation of social conditioned place preference in male C57Bl6/J mice [23]. In California mice and prairie voles, social stressors reduce oxytocin immunoreactivity and oxytocin receptor (OTR) binding in the NAc, corresponding with reductions in social approach [24, 25]. Conversely, in the bed nucleus of the stria terminalis (BNST) stress increases the activity of oxytocin producing cells [26, 27], and inhibition of OTR in the BNST prevents stress-induced decreases in social approach and increases in social vigilance in female California mice [24]. Social vigilance is defined as orienting toward an unfamiliar individual without approaching [24], and resembles responses by rats confronted with a predator [28] and stress-induced stretch-attend responses in Syrian hamsters [29]. Social vigilance has also been observed in female C57Bl6/J mice exposed to social stress [30]. In humans, a combination of increased social vigilance and reduced social approach is characteristic of behavioral inhibition, a temperament that is a strong predictor for the development of social anxiety disorders [31–34]. Previously, we showed that treatment with an OTR antagonist increased social approach and decreased social vigilance [24] in stressed female California mice if administered either systemically or site-specifically into the BNST. A key question is what the specific contributions of NAc OTR are on stress-induced changes in social approach and social vigilance. Specifically, does activation of NAc OTR have distinct effects on these behaviors, or are social approach and social vigilance universally inversely related?

An additional layer of complexity stems from the distinct signaling pathways and cellular processes that can be activated by OTR coupling with different G-protein subunits: excitatory subunit Gq and inhibitory subunit Gi [35]. Biased agonists that selectively activate distinct signaling pathways by promoting G-protein coupled receptor coupling to selective G-protein subunits [36] have been proposed as novel treatment options for various mood and pain disorders [37, 38]. Some evidence exists for the ability of OTR to reduce general anxiety-like behavior in male Sprague-Dawley rats through Gq specific signaling pathways [39], suggesting that agonists that selectively activate OTR-Gq signaling could have potential as novel therapeutics for anxiety disorders. In contrast, recent data showed that Atosiban, a biased agonist that induces OTR-Gi signaling and inhibits OTR-Gq [35], had analgesic effects in the spinal cord in male Wistar rats [40]. Here, we test the extent to which biased agonists with distinct signaling properties can act in the NAc core to modulate social approach and social vigilance. First, we examined the cellular distribution of *Oxtr* mRNA and assessed whether social stress impacted *Oxtr* expression in the NAc. Next, we used L-368,899 (OTA, an unbiased OTR antagonist [24]) to block OTR signaling through both Gq and Gi pathways in unstressed mice. We then used Atosiban to block OTR-Gq signaling while activating OTR-Gi signaling in unstressed mice. Last, we used Carbetocin, which induces OTR-Gq signaling without engaging OTR-Gi [41], in stressed mice in order to test the extent to which OTR-Gq signaling can reverse stress-induced deficits in social approach and increases in social vigilance. Our results suggest that social approach and social vigilance are controlled by distinct but overlapping mechanisms.

## Methods and materials

For full details see Supplementary “Materials and Methods”.

### Animals

All studies on California mice (*Peromyscus californicus*) were in accordance with the NIH Guide for the Care and Use of Laboratory Animals and approved by the Institutional Animal Care and Use Committee at the University of California, Davis. Mice were housed in clear polypropylene cages on a 16 L:8D light cycle. No systematic biases in the distribution of estrous stage across treatment groups were detected across experiments (Table S1).

### Fluorescent in situ hybridization (FISH) and quantitative real-time PCR (qPCR)

We used ACDBio RNAscope methods to perform FISH using custom primers based on sequences in California mice to assess colocalization of *Oxtr* in *Gad1* cells. Next, qPCR was used to assess the effects of social defeat on *Oxtr* expression in the NAc. For primer sequences used see Table S2.

### Cannula placement and site-specific injections of OTR ligands

Male and female mice were implanted with bilateral guide cannulas (Plastics One, Roanoke, VA) aimed at the NAc core using a California mouse brain atlas (brainmaps.org, anteroposterior: +0.51, mediolateral: ±1.5, dorsoventral:+6.0) [24]. Depending on the experiment, mice were randomly assigned to receive bilateral 0.2 μl infusions of either artificial cerebrospinal fluid (aCSF), OTA (1 μg; L-368,899 unbiased OTR antagonist), Atosiban (1 ng; OTR-Gq antagonist, OTR-Gi agonist), or Carbetocin (1 μg; OTR-Gq agonist, OTR-Gi antagonist). See Supplementary methods for details on dosage selections. A social interaction test was run 30 min after infusion. Mice receiving Carbetocin were run through social defeat stress 1 week prior to surgery. Histology was used to confirm injection placement.

### Social defeat stress

For experiments 1 and 4, mice were randomly assigned to social defeat (placed into home cage of an aggressive same-sex resident) or control handling for three consecutive days as previously described [42, 43]. See Supplementary methods for full details.

### Social interaction test and social vigilance

Social interaction testing was performed as previously described [42, 43]. Mice were placed in an open arena for 3 min (open field). Next, an empty wire cage was placed in the arena and the time spent within 8 cm of the cage (interaction zone) was scored for 3 min (acclimation phase). For the last phase, an unfamiliar same-sex mouse was placed into the wire cage and time spent near the interaction zone was scored for 3 min (interaction phase). We define time spent in the interaction zone with a target mouse as social approach. Social vigilance was scored during the interaction phase by recording the amount of time the focal mouse spent with its head oriented toward the target mouse while outside the interaction zone [24, 44]. See Supplementary Video S1 for representative examples.

### Statistical analyses

All statistical analyses were performed using R statistical software. Normality of data was assessed using Shapiro-test. A Fligner-Killeen test was used to assess homogeneity of variance. Two-way ANOVA was used to analyze qPCR data as well as behavior measures in experiment three and four. Three-way ANOVA was used to analyze behavioral data in experiment two. For data that did not follow a normal distribution (vigilance), data were square root transformed to normalize prior to ANOVA testing. For ANOVA analyses that revealed significant interaction effects, pairwise comparisons were used to detect differences between groups.

## Results

### Experiment 1: *Oxtr* is expressed in inhibitory neurons and expression is reduced by social stress

**Fig. 1:**
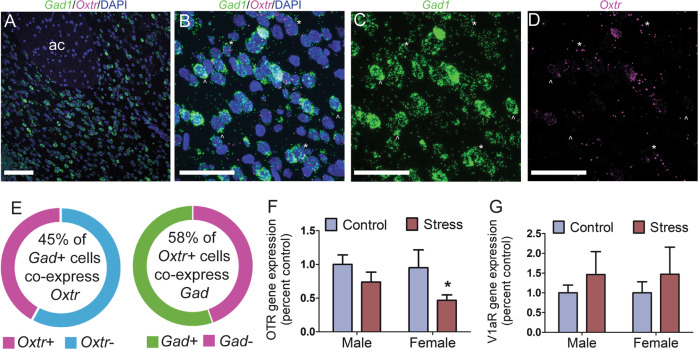
*Oxtr* cell-type localization and stress-induced changes in gene expression. Representative photomicrographs of *Gad1*/*Oxtr*/DAPI fluorescent in situ hybridization in NAc (**a**–**d**). *Gad1*/*Oxtr*/DAPI overlay (**b**). *Gad*+ cells (**c**). *Oxt*+ cells (**d**). Circle chart representing percentage of *Oxt* +/*Gad*+ cells present in the NAc (**e**). *Otxr* (**f**) and *V1aR* (**g**) gene expression in the NAc following stress in males and females. *p < 0.05 vs. control. Group N’s: control/male: 12, control/female: 13, stress/male: 10, stress/female: 12. Scale bar 100 µm (**a**), 40 µm (**b**–**d**).

To determine whether *Oxtr* is expressed locally in inhibitory neurons within the NAc core, we performed in situ hybridization using probes directed against *Oxtr* or *Gad1* (Fig. 1a–d). *Oxtr* was expressed in just over half (58.0%) of all *Gad1* nuclei, indicating local expression in inhibitory neurons (Fig. 1e). To quantify the effects of social defeat stress on *Oxtr* expression in males and females we used real-time PCR. Overall, social defeat reduced *Oxtr* expression in NAc punch samples (Fig. 1f, stress effect: *F*_1,43_ = 4.6, *p* = 0.038). However, planned comparisons only detected a significant stress effect in females (Fig. 1f). No effects of stress were observed in *V1aR* expression (Fig. 1g).

### Experiment 2: unbiased OTR antagonist in the NAc reduces social approach without increasing social vigilance

**Fig. 2:**
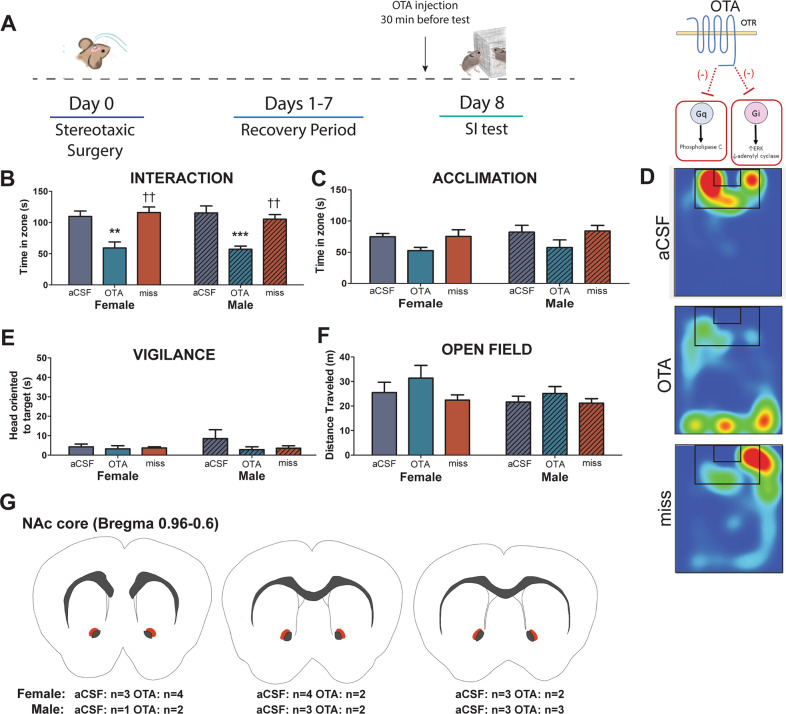
OTA infusion in the NAc decreases social approach without inducing social vigilance in male and female California mice. Timeline of experiment and schematic of mechanism of action for L-368,899 (OTA) (**a**). OTA infusion in the NAc reduced social approach when a target was present (**b**) but not when a target was absent (**c**) compared with mice infused with aCSF or mice with misplaced infusion posterior to NAc of either treatment. Representative heatmaps for the interaction phase showing reduced time spent in the interaction zone while a target was present in mice receiving OTA, but not aCSF or misplaced injection. Heatmaps are of a representative mouse (**d**). There were no differences in vigilance behavior (**e**) or general locomotion during an open field phase (**f**). Schematic representing injection sites (orange shading) of successful cannula placement (**g**). **p < 0.01 vs. aCSF, ***p < 0.001 vs. aCSF, ^††^*p* < 0.01 vs. OTA. Group N’s: female/OTA: 7, male/OTA: 8, female/aCSF: 7, male/aCSF: 10, female/miss: 7, male/miss: 13.

To test the effects of OTR antagonism in the NAc on behavior, unstressed males and females were given an infusion of the unbiased OTR antagonist L-368,899 (OTA) and tested in a social interaction test (Fig. 2a). In both males and females, correctly placed infusions of OTA reduced time spent in the interaction zone with the target mouse (treatment*hit interaction, *F*_1,48_ = 9.311, *p* = 0.039, Fig. 2b, d, g). No significant effects of sex (main effect of sex, *F*_1,48_ = 0.152, *p* = 0.69) or stress interaction effects (treatment*hit*sex, *F*_1,48_ = 0.026, *p* = 0.87) were found. In females OTA infusions in the NAc reduced time spent in the interaction zone with the target mouse compared with aCSF (*p* = 0.0014, Fig. 2b) or infusions made outside the NAc (both *p'*s < 0.01, Fig. 2b). The same result was seen in males, where those infused with OTA in the NAc showed more social approach than those that received aCSF (*p* < 0.001, Fig. 2b) or infusions made outside of the NAc (both *p*'s < 0.01, Fig. 2b). No differences were observed during the acclimation phase when the target was absent (Fig. 2c). Importantly, there was no effect of OTA infusions on social vigilance in males or females (Fig. 2e), even though this study was sufficiently powered to detect effect sizes based on previous publications (power = 0.91). In addition, there were no differences in locomotion during the open field (Fig. 2f), and no sex differences were observed (Fig. 2b–f). In summary, these results show that reduced social approach can be dissociated from increased social vigilance (Supplementary Video 1).

### Experiment 3: social approach in female California mice is reduced by Atosiban

**Fig. 3:**
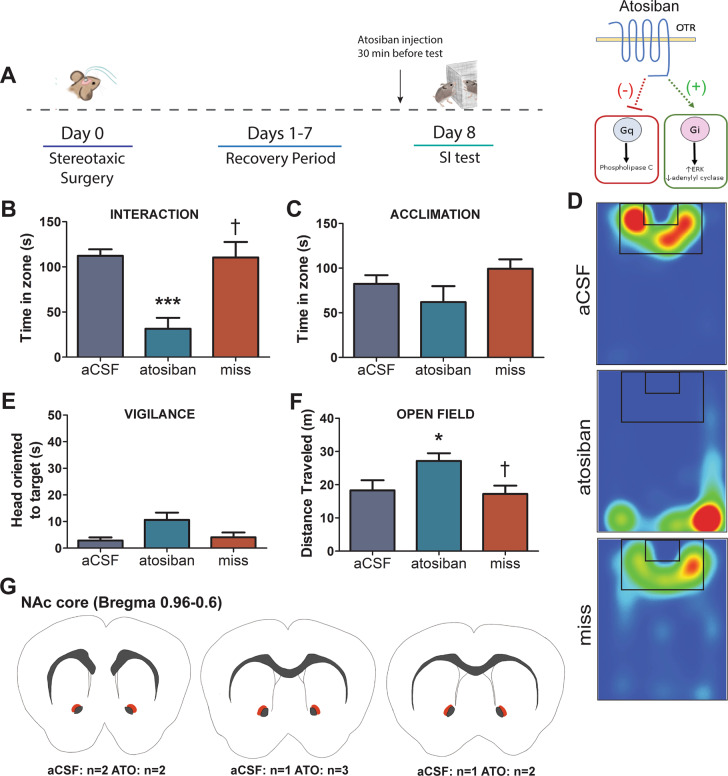
Atosiban infusion in the NAc decreases social approach without increasing social vigilance in unstressed female California mice. Timeline of experiment and schematic of mechanism of action for Atsoiban (**a**). Atosiban infusion in the NAc of unstressed female mice decreased social approach when a target was present (**b**) but not when a target was absent (**c**) compared with unstressed females infused with aCSF or mice with misplaced infusion posterior to NAc of either treatment. Representative heatmaps for the interaction phase showing reduced time spent in the interaction zone in unstressed females receiving Atosiban, but not aCSF or misplaced treatment. Heatmaps are of a representative mouse (**d**). Atosiban infusion in the NAc did not induce a vigilance phenotype (**e**). Atosiban infusion increased general locomotion during an open field phase (**f**). Schematic representing injection sites (orange shading) of successful cannula placement (**g**). ***p < 0.001 vs. aCSF, *p < 0.05 vs. aCSF, ^†^*p* < 0.05 vs. Atosiban. Group N’s: Atosiban: 7, aCSF: 4, miss: 6.

To investigate downstream mechanisms of NAc OTR on social approach unstressed females were treated with Atosiban, a biased OTR ligand that selectively inhibits Gq signaling while activating Gi signaling (Fig. 3a). Atosiban infusions made in the NAc reduced time spent in the interaction zone with the target (treatment*hit interaction, *F*_1,13_ = 13.93, *p* = 0.002; Fig. 3b, d, g) compared with females treated with aCSF (*p* < 0.001, Fig. 3b) or females with injections made outside the NAc (both *p's* < 0.05, Fig. 3b). No differences were observed during the acclimation phase when the target was absent (Fig. 3c). Vigilance was unaffected by treatment; all females showed low levels of vigilance (Fig. 3e). Females treated with Atosiban in the NAc showed significantly more general locomotion during an open field phase (treatment*hit interaction, *F*_1,13_ = 5.42, *p* = 0.036, Fig. 3f). Females treated with Atosiban in the NAc showed greater distance traveled during the open field phase compared with females treated with aCSF (*p* = 0.034) and misplaced injections (both *p*'s < 0.05).

### Experiment 4: effects of social defeat on social approach and vigilance are reversed by Carbetocin

**Fig. 4:**
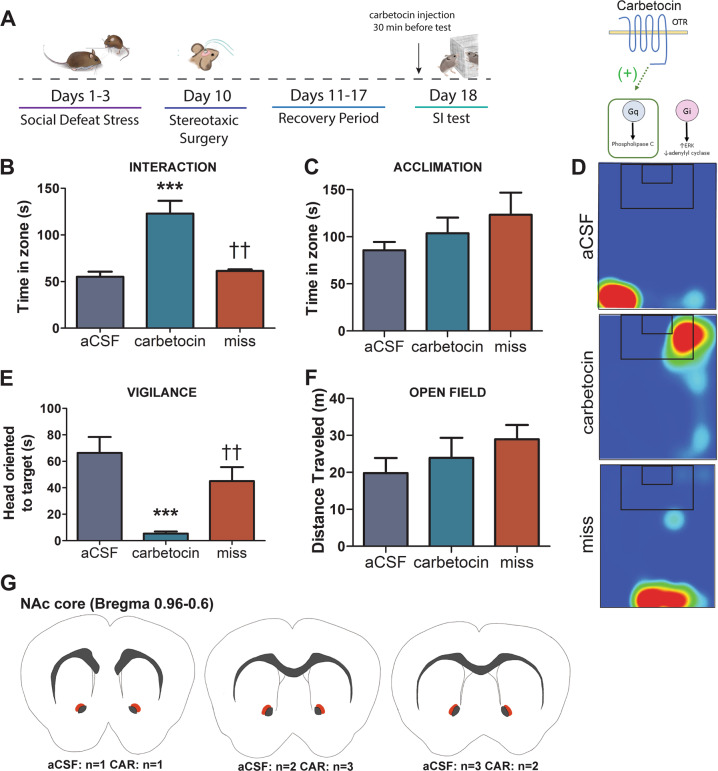
Carbetocin infusion in the NAc reduces effects of stress on social approach and social vigilance in female California mice. Timeline of experiment and schematic of mechanism of action for Carbetocin (**a**). Carbetocin infusion in the NAc of stressed female mice increased social approach when a target was present (**b**) but not when a target was absent (**c**) compared with stressed females infused with aCSF or mice with misplaced infusion posterior to NAc of either treatment. Representative heatmaps for the interaction phase showing increased time spent in the interaction zone while a target was present in stressed females receiving Carbetocin, but not aCSF or misplaced injections. Heatmaps are of a representative mouse (**d**). Carbetocin infusion in the NAc reduced stress-induced vigilance (**e**). No group differences in general locomotion during an open field phase were observed (**f**). Schematic representing injection sites (orange shading) of successful cannula placement (**g**). ***p < 0.001 vs. aCSF, ^††^*p* < 0.01 vs. Carbetocin. Group N’s: Carbetocin: 6, aCSF: 6, miss: 4.

To further test the downstream mechanisms of NAc OTR on social approach, stressed females were given an infusion of Carbetocin, a biased OTR-Gq agonist (Fig. 4a). Carbetocin administration into the NAc significantly increased time spent in the interaction zone with the target (interaction*hit interaction, *F*_1,12_ = 6.86, *p* = 0.022, Fig. 4b, d, g) compared with mice treated with aCSF or mice receiving injections placed outside the NAc (all *p'*s < 0.01, Fig. 4b). No differences were observed when the target was absent during the acclimation phase (Fig. 4c). Contrary to the previous studies, Carbetocin infusions made in the NAc reduced vigilance (treatment*hit interaction, *F*_1,12_ = 4.46, *p* < 0.05; Fig. 4e) compared with mice treated with aCSF (*p* < 0.001, Fig. 4e) or mice receiving injections made outside of the NAc (both *p*'s < 0.01, Fig. 4e). No differences were seen in general locomotion during an open field phase (Fig. 4f).

## Discussion

Across three experiments, using site-specific injections of two different OTR antagonists, L-368,899 and Atosiban, we show that reduced OTR signaling within the NAc reduces social approach without increasing social vigilance. First, the unbiased OTR antagonist L-368,899 (OTA) reduced social approach but did not increase social vigilance in both males and females. Second, despite its OTR-Gi agonism properties, inhibition of OTR-Gq signaling with Atosiban had similar effects on social approach, suggesting that OTR-Gq in the NAc is necessary to promote social approach. Consistent with this hypothesis, Carbetocin, which selectively induces OTR-Gq signaling, increased social approach in stressed females. Importantly, Carbetocin also inhibited social vigilance. Taken together, these results suggest that activation of OTR-Gq signaling in the NAc can promote social approach, but that inhibition of OTR-Gq signaling within the NAc is insufficient to induce social vigilance. These key results show distinct neural regulation of social approach and vigilance.

Social stress reduces social approach in many rodent species [24, 25, 44–46], and this behavioral change often co-occurs with increases in social vigilance [24, 30, 44]. In male C57Bl6/J, Wistar rats, and prairie voles, stress-induced decreases in social approach can be reversed by oxytocin infusion in the NAc [25, 47]. However, it was previously unclear whether OTR in the NAc also influences social vigilance. Using OTA we showed that blocking OTR in the NAc does not impact social vigilance in unstressed males or females. However, an additional layer of complexity is that OTR can activate multiple G-protein subunits (both Gq and Gi), and thus can activate multiple signaling pathways [35, 48]. This is significant because the actions of a receptor on behavior may depend on which signaling pathways are activated [38, 49, 50]. Relatively little is known about how biased OTR signaling modulates behavior. In one study, activation of OTR-Gq signaling via central Carbetocin treatment increased time spent in the open arms in the elevated plus maze in male Wistar rats [39]. Here, we show that intra-NAc infusion of Atosiban reduces social approach in unstressed females while Carbetocin increases social approach in stressed females. Together, these findings support the hypothesis that OTR-Gq coupling in the NAc promote social approach. Unstressed males and females treated with OTA also showed reduced social approach but did not display increases in social vigilance. The consistent effects of the OTR antagonists on behavior suggests that circuits modulating social approach and social vigilance can be dissociated from each other.

An unexpected result was that treatment with Atosiban in the NAc selectively increased locomotion during an open field test. Atosiban was the only ligand used that induces OTR-Gi coupling and was also the only ligand to increase locomotion in the open field phase. Interestingly, dopamine-induced locomotion can also be mediated by activation of inhibitory signaling pathways. Chemogenetic activation of neurons expressing dopamine D2 receptors (which induce Gi/Go coupling [51]) in the NAc increased locomotor behavior in male C57BL/6J mice while chemogenetic activation of neurons expressing D1 receptors (which induce Gs coupling [51]) did not [52]. These data correspond with studies using the D2 agonist quinpirole, which increases locomotion when administered site-specifically in the NAc of male Sprague-Dawley and Long-Evans rats [53, 54]. It is possible that selective activation of OTR-Gi coupling may induce similar signaling cascades as D2 receptors, which could explain why locomotor behavior was altered by Atosiban. An important consideration is that Atosiban and Carbetocin have the ability to act as antagonists at vasopressin receptor 1A (V1aR) [41, 55]. However, the behavioral effects we report are unlikely to be mediated by V1aR because previous work showed very low levels of V1aR binding in the NAc, and that infusion of selective V1aR antagonists into the NAc had no effect on social approach or locomotor behavior in female California mice [56]. In addition, although L-368,899 has the ability to act as an antagonist at vasopressin receptor 1B (V1bR) [57], levels of V1bR have been reported to be either very low or nonexistent in the NAc of male rats [58–60], therefore we do not expect our findings to be a result of V1bR antagonism. It is important to note that unlike oxytocin, Carbetocin has been shown to promote OTR internalization without recruiting β-arrestins, through an undefined endocytic pathway [41]. The failure to recruit β-arrestins, or the use of an alternative endocytic pathway, which could engage either selective signaling intermediates [61] or molecules [62], could contribute to Carbetocin having specific signaling outcomes in circuits involved in regulating social vigilance. Identifying signaling pathways activated by Carbetocin effects in the NAc will be an important question for future studies.

We also observed stress-induced decreases in *Oxtr* gene expression in females, which corresponds with previous studies showing reduced OTR binding and reduced social approach in the NAc after defeat in female California mice [24, 25]. In addition, FISH analyses show that in adult California mouse NAc, 58% of *Oxtr* positive cells coexpress *Gad1*. The remaining 42% of *Oxtr* positive cells are likely to be other neuronal cell-types, such as astrocytes and microglia, which express *Oxtr* [63] but do not contain detectable levels of *Gad1* [64]. While in male C57Bl6/J mice social place preference is mediated by OTR acting through a pre-synaptic mechanism [23] in juvenile but not adult mice [65], our FISH data suggest the possibility that locally expressed post-synaptic OTR could be important for modulating behavioral effects of oxytocin in the NAc. It is also important to consider that social approach and social place preference may reflect different behavioral processes, as the place preference assay involves a learning component. Future studies assessing the impact of pre- and post-synaptic NAc OTR, especially those utilizing mice across ages, will be needed to fully understand how oxytocin action in the NAc modulates social preference and approach [66]. It will also be interesting to determine cell-type specific roles of *Oxtr* in the NAc on social approach and reward.

Overall our results suggest that OTR in the NAc modulate social approach primarily through an OTR-Gq mechanism, consistent with previous findings in male Wistar rats showing reduced anxiety-like behavior following i.c.v. treatment with Carbetocin [39]. In addition, we find that a lack of OTR-Gq signaling in the NAc reduces social approach independently of effects on social vigilance. Taken together these results suggest that OTR sensitive circuits controlling social approach and vigilance are distinct but overlapping, and that induction of social vigilance by oxytocin requires the recruitment of additional circuits. An important question for future studies is whether baseline sex differences exist in OTR coupling to Gq or Gi subunits, and whether OTR coupling to subunits is different in the NAc than in other regions. In addition, it will be interesting to test whether stress exposure impacts this coupling and contingent signaling mechanisms. Future studies examining these topics will be instrumental for gaining a better understanding of how OTR-Gq and Gi signaling mechanisms influence anxiety-like behavior.

## Funding and disclosure

Supported by R01MH121829 and R01MH103322 to BCT. The authors declare no competing interests.

## Supplementary information

Supplementary Table 1: Estrous stage for experiments 2, 3 and 4.

Supplementary Table 2: Transcript sequences used for designed qPCR primers.

Supplementary Methods

Supplementary Video 1: Social approach, social avoidance, and social vigilance phenotypes.
