# Supplementary material for: Social approach and social vigilance are differentially regulated by oxytocin receptors in the nucleus accumbens
Source: Neuropsychopharmacology. 2020 Mar 20;45(9):1423–30. doi: 10.1038/s41386-020-0657-4 (PMC7360746; doi:10.1038/s41386-020-0657-4)
Supplement: Supplementary file 1 — Supplementary Table 1: Estrous stage for experiments 2, 3 and 4. [file 41386_2020_657_MOESM1_ESM.docx]

**Supplementary Table 1: Estrous stage for experiments 2, 3 and 4.**

| Experiment 2 | Main effect estrous | Estrous*treatment | Pearson’s Correlation  (estrous x treatment) |
| --- | --- | --- | --- |
| Social Approach | p=0.2 | p=0.9 | p=0.4 |
| Social Vigilance | p=0.1 | p=0.5 | p=0.2 |
| Experiment 3 and 4 |  |  |  |
| Social Approach | p=0.1 | p=0.1 | p=0.3 |
| Social Vigilance | p=0.3 | p=0.7 | p=0.2 |
