# Supplementary material for: Social approach and social vigilance are differentially regulated by oxytocin receptors in the nucleus accumbens
Source: Neuropsychopharmacology. 2020 Mar 20;45(9):1423–30. doi: 10.1038/s41386-020-0657-4 (PMC7360746; doi:10.1038/s41386-020-0657-4)
Supplement: Supplementary file 2 — Supplementary Table 2: Transcript sequences used for designed qPCR primers. [file 41386_2020_657_MOESM2_ESM.docx]

**Supplementary Table 2: Transcript sequences used for designed qPCR primers.**

| Gene ID | Forward Sequence | Reverse Sequence |
| --- | --- | --- |
| *Oxtr* | GCCCTTGACGCCTTTCTTCT | TTCCTTGGGCGCATTGAC |
| *B2m* | TCTAGTGGGAGGTCCTGTGG | TGCGTTAGACCAGCAGAAGG |
