# Supplementary material for: Social approach and social vigilance are differentially regulated by oxytocin receptors in the nucleus accumbens
Source: Neuropsychopharmacology. 2020 Mar 20;45(9):1423–30. doi: 10.1038/s41386-020-0657-4 (PMC7360746; doi:10.1038/s41386-020-0657-4)
Supplement: Supplementary file 3 — Supplementary Methods [file 41386_2020_657_MOESM3_ESM.docx]

*Supplementary Methods*

**Animals and housing conditions**

Adult male and female California mice (*Peromyscus californicus*), 3–6 months old, were bred in our laboratory colony and housed in same-sex groups of two to three per cage on Sani-Chips bedding with cotton nestlets in clear polypropylene cages. Mice were kept on a 16-hour light/8-hour dark cycle (lights on at 23:00 h) with Teklad 2016 food (Harlan, Hayward, CA, USA) and water provided ad libitum. All procedures were in accordance with the NIH Guide for the Care and Use of Laboratory Animals and approved by the Institutional Animal Care and Use Committee at the University of California, Davis. Mice were euthanized after behavior testing by 5% isoflurane administration followed by transcardial perfusion or rapid decapitation. Estrous cycle was assessed post-mortem to avoid disrupting behavior [64]. No systematic biases in the distribution of estrous stage across treatment groups were detected across experiments (Supplementary Table 1).

**Social Defeat Stress**

Mice were randomly assigned to social defeat or control handling for 3 consecutive days. Mice assigned to social defeat were placed in the cage of an aggressive same-sex mouse [41]. Each episode lasted 7 min or until the resident attacked the focal mouse 7 times, whichever occurred first. Control mice were placed in a clean cage for 7 min. Immediately following defeat or control conditions mice were returned to their home cage [41,42].

**Quantitative Real-Time PCR**

RNA was extracted from punch samples of NAc dissected from fresh frozen tissue using RNAeasy Mini Kits (Qiagen) and QIAzol as a lysis reagent (Qiagen). Reverse transcription was performed using iScript (BioRad). All sequences were amplified using SYBR green chemistry on an Applied Biosystems ViiA7 instrument. Specific forward and reverse primers for *Oxtr* (Genbank accession: MN265350): and *B2m* (Genbank accession: XM_006995122) mRNA were designed based on California mouse sequence (see Supplementary Table 2). There were no differences in cycle thresholds between groups for *B2m*. For each sample *Oxtr* mRNA was normalized to *B2m* expression.

**Fluorescent In-Situ Hybridization**

Fluorescent in situ hybridization was performed using ACDBio RNAscope multiplex fluorescence methods [65]. We designed probes to detect *Oxtr* (Genbank accession: MN265350) or *Gad1* (Genbank accession: MN265351) using full length California mouse mRNA sequences (see Supplementary Table 2). Brains from adult male (n=2) and female (n=2) mice were flash frozen and cut at 20 μm. Sections were fixed in chilled 10% neutral buffered formalin for 15 min and then dehydrated in a series of ethanol baths (50%, 70%, 100%). Next protease IV was applied to each section for 30 min and then washed in phosphate buffered saline (PBS). One set of sections was probed for *Oxtr*/*Gad1* and a second set of sections was probed with Oxtr/vGlu2. Probes were diluted 1:50 and applied to slides for 2 h at 40° C. Slides were then rinsed in wash buffer and then incubated sequentially in amplification buffer (AMP) 1 for 30 min at 40° C. Slides were rinsed in wash buffer and the incubated in AMP 2 for 30 min at 40° C. Slides were developed in Cy3 (Oxtr) for 15 min and then fluorescin (*Gad1*) for 30 min. Slides were coverslipped in Vectashield with DAPI.

We collected 20x confocal z-stack images. Images were acquired at 9 pixels per μm as X mm z-stacks with a step size of y mm. For colocalizations analyses, *Oxtr* nuclei were counted and then determined to be *Gad1* positive or negative.

**Cannula placement and site-specific injection of OTR ligands**

Male and female mice were implanted with bilateral guide cannulas (Plastics One, Roanoke, VA) aimed at the nucleus accumbens core (NAc) (anteroposterior: +0.51, mediolateral: ±1.5, dorsoventral:+6.0) [25]. After 1 week of recovery, mice were randomly assigned to receive bilateral 0.2μl infusions of specific OTR ligands. In experiment 2, males and females were infused with either artificial cerebrospinal fluid (aCSF) or a 1μg dose of OTA (full OTR antagonist; Tocris, 2641) into the NAc core [25]. 30 min following each animal was run through a social interaction test (Fig. 2A). In experiment three, females were randomly assigned to receive a bilateral 0.2μl infusion of either aCSF or a 1ng dose of Atosiban (OTR-Gq antagonist, OTR-Gi agonist; Tocris, 6332) into the NAc core. 30 min following injection a social interaction test was run 30m later (Fig. 3A). In experiment 4, females experienced 3 days of either social defeat stress or control handling 1 week prior to surgery (Fig. 4A). Females were then randomly assigned to receive a bilateral 0.2μl infusion of either aCSF or a 1μg dose of Carbetocin (OTR-Gq agonist, OTR-Gi antagonist; Tocris, 4852) into the NAc core. 30 min following infusion, females were run through a social interaction test (Fig. 4A). The dose for Carbetocin is based on previous behavioral studies as well as in vitro work showing that the efficacy of oxytocin for inducing OTR-Gq coupling is 100-fold higher than Carbetocin. The dose of Atosiban is based on previous showing that the affinity for Atosiban for OTR is in the nM range, similar to oxytocin [47].

**Social Interaction Test and Social Vigilance**

For social interaction testing, each focal mouse was introduced into the open field (89 × 63 × 60 cm) for 3 min (open field phase) [41,42]. Total distance traveled was recorded to assess locomotor behavior (Anymaze, Stoelting). Next, an empty wire cage was introduced into the arena and the time that the focal mouse spent within 8 cm of the cage (interaction zone) was recorded for 3 min. Finally, an unfamiliar intact same-sex mouse was placed into the wire cage for 3 min (interaction phase) and the time spent in the interaction zone was recorded. Social vigilance was scored during the interaction phase by recording the amount of time the focal mouse spent oriented towards the interaction zone [25,43]. Mice could be moving or stationary while engaging in social vigilance.

**Statistical Analyses**

All statistical analyses were performed using R statistical software. Normality of data was assessed using Shapiro-test. A Fligner-Killeen test was used to assess homogeneity of variance. Two-way ANOVA was used to analyze qPCR data as well as behavior measures in experiment 3 and 4. Three-way ANOVA was used to analyze behavioral data in experiment 2. For data that did not follow a normal distribution (time spent oriented towards the target, or vigilance), data were square root transformed to normalize prior to ANOVA testing. For ANOVA analyses that revealed significant interaction effects, pairwise comparisons were used to detect differences between groups.
